# Four Successive Ruptures of the Uterus in the Same Patient with Favorable Termination

**Published:** 1877-08

**Authors:** J. M. Rose

**Affiliations:** West Winfield, N. Y.


### FOUR SUCCESSIVE RUPTURES OF THE UTERUS IN THE SAME PATIENT WITH FAVORABLE TERMINATION.

By Dr. J. M. Rose, of West Winfield, N. Y.

Mrs. P. Da—, native of Ireland, age 32 years; has resided in this country about twenty years. She is the mother of two children, the oldest four and the youngest two years of age. Mrs. D. was taken with labor pains, June 1st, 1869. The pains were regular, the head presented, the os dilated well, and everything promised a favorable termination of the case within a few hours.

After she had been in labor about five hours, she had quite a severe pain, and complained more than usual. This pain was followed by fainting, with general prostration, and in a short time with great tenderness over the whole abdomen. Upon introducing my hand into the vagina, I could feel nothing of the child. Suspecting a rupture of the uterus, and thinking that the patient would die, I sent for my friend, Dr. E. King. After consultation we concluded to get the fœtus away if possible. I gave the patient a dose of opium and some milk punch. I then proceeded to deliver her. I passed my hand through the os, and finding a rupture passed my hand through the opening into the cavity of the abdomen. I succeeded in finding the feet, brought them down and delivered a dead child in about twenty minutes, together with the after-birth, which was also in the cavity of the abdomen. She bore the operation quite well. I ordered her to take an eighth of a grain of morphine in connection with two grains of quinine every four hours, unless she rested, with beef tea and milk punch for food. I also gave half a drop of fluid extract of veratrum viride every four hours.

June 2d. I found the patient better than I expected; pulse, 80; temperature, nearly normal. Continued treatment.

June 3d. Pulse, 80; more irritable, bowels very tender to the touch. Continued treatment.

June 4th. In the morning, pulse, 90; in the evening, 120. Bowels, tympanitic; vomiting, can retain but very little nourishment. She sleeps but little; pain not very severe unless she is moved, or pressure is made on her bowels. Stopped veratrum viride.

June 5th. Pulse, 120, morning and evening; vomiting; tongue red; gave syrup of chloroform, which stopped the vomiting.

June 6th. Pulse, 110, morning and evening; bowels less tender, and have moved once. Continue morphine and quinine.

June 7th. Pulse, 100 in the morning, 110 in the evening; rested quite well. Skin moist the most of the time.

June 8th. Pulse, 100. Calls for food; wants meat and potatoes. She has lived principally upon milk punch and beef tea up to this time.

June 9th. Pulse, 100; tongue red, symptoms favorable. Treatment continued.

June 10th. Pulse, 95; bowels moved four times. There is considerable pain. Increased the amount of morphine.

June 11th. Pulse, 95; bowels quiet; is better.

June 13th. Pulse, 90; bowels quick, no tympanitis; tongue appears better.

June 15th. Continues to improve; can turn in bed without help.

June 17th. Still improving; pulse, 90; tongue moist and not so red. Continue quinine, with the addition of morphine at evening.

June 20th. Improving; patient wanted to get up. I told her to wait.

June 24th. Pulse, 85; skin natural; sat up a short time.

June 30th. Pulse, 80; tongue looks quite natural; bowels tender to pressure; can move about by being very careful. Continue tonics, iron and bark, with careful diet. In a short time she was at work.

I was again sent for to attend Mrs. D—, in confinement,

April 1st, 1872. I found her in good spirits, thinking that she was going to get along all right. The pains went along as usual for three or four hours when they suddenly stopped, and were followed by great prostration and tenderness of the bowels. Suspecting that the uterus had again ruptured, I made an examination and found that the head had receded, and passing my hand into the uterus I found that my suspicion was correct. I proceeded to deliver as before, by passing my hand through the rent in the womb and bringing down the feet. I delivered her in a few minutes. She bore the operation very well.

April 2d. I found the patient quite comfortable; gave anodynes with supporting treatment.

April 3d. Bowels very tender; pulse 100; temperature 99; gave aconite in addition.

April 4th. Pulse 100; temperature 100; bowels tympanitic.

April 6th. Pulse 110; temperature 101; vomiting; bowels quite tender, with purulent discharges from the womb.

April 9th. Pulse 110; temperature 100. Is taking light food—beef tea and milk punch.

April 15th. Pulse 100; temperature 98; appetite good; symptoms quite favorable; was lying in bed and taking care of her two children sick with scarlet fever. She improved gradually, and in about a month was doing light work. In connection with the use of the anodynes and tonics, I applied turpentine to the bowels.

It may be proper to state that the long intervals between my visits, were due to the state of the roads, which were in such a condition as to render it impossible for me to visit her as often as I thought she needed.

On May 18th, 1874, Mrs. D——— was again taken with labor pains, which lasted about two hours and stopped suddenly, followed by feelings of faintness, with tenderness over the abdomen. A physician had been sent for, who told her that the pains had stopped, and that perhaps they would not come on in a week or more. On the strength of his assertion, they waited until the morning of the 20th, when they sent for me. Upon making an examination, I found the os partially dilated, so that I could introduce my hand and pass it into the womb, when I discovered that it was again ruptured. I told her husband that there was great danger of her not recovering, when he said that I must wait before doing anything until the arrival of the priest, as they had sent for him and he would be there in a few hours. I left, telling him that I would be back at 1 o'clock p. m., and advised him to send for Dr. King to meet me at that time. The doctor came, and confirmed my diagnosis, and after the priest had got through with the patient, we proceeded to deliver her. She had become so tender that it was necessary to give chloroform. Dr. King give chloroform. As soon as she became quiet, I introduced my hand and brought away the placenta, which lay in the abdominal cavity near the opening. I then passed my hand again; the head was in the lower portion of the bowels. I carried my hand up and got hold of the feet, and delivered her in a few moments.

The child had been dead so long that the skin would slip off upon handling.

After delivering the child, I introduced my hand again. I could pass it up to the upper portion of the abdominal cavity, and move it about without encountering any adhesions or obstacles of any kind. The rent in the womb was transverse and in its posterior portion; it felt as though half of the womb had been cut off with a knife. The womb had contracted to about the size of the two fists. None of the contents of the abdomen passed through the rent. She bore the operation very well.

May 21. Patient apparently doing well; takes beef tea, milk punch and opium.

May 22. Pulse 110; bowels tympanitic; fever, with darting pains; great tenderness. Rested some last night.

May 23. Pulse 120; temperature 100. She had been vomiting, and had had three movements from the bowels, which caused her to be in great pain. Continued opium and quinine, with gelseminum every four hours.

May 24. Pulse 120; temperature 102. Had a diarrhœa; bowels moved quite often. Added subnitrate of bismuth to powders.

May 25th. Diarrhœa continued. She had twenty or thirty movements. Temperature, 100; pulse 120,

May 27th. Pulse, 107; temperature, normal. Some appetite; bowels moved four times. Continue bismuth and opium treatment.

May 29th. Pulse, 94; bowels moved twice. Continued treatment.

June 1st. Pulse, 90; bowels regular. Sat up an hour yesterday. All favorable.

Since writing the above, Mrs. D——— has been delivered of a living child, after another rupture of the womb.

She was taken sick about one o'clock in the morning of February 28th, 1876. I saw her at a quarter past twelve the same day. She had been having regular pains for about two hours. About twenty minutes before I saw her, she had a pain, and the waters broke; she said she felt a great movement of the child,—she felt it pressing up under her ribs. I made an examination, and could just reach one foot with the finger. Introduced my hand and brought down the feet, and delivered a living child as soon as possible. The delivery was accomplished in about ten minutes, before the placenta was detached from the womb. There was strong pulsation in the cord after the delivery of the child.

29th. Mrs. D——— is quite comfortable. Pulse, 85; not much fever. She is suffering no more than is usual after an ordinary delivery.

30th. Doing well. Pulse, about 80; temperature natural. No great tenderness of bowels. A little bloody discharge from the vagina.

March 5th. Mrs. D——— is doing finely.

